# High accuracy barrier heights, enthalpies, and rate coefficients for chemical reactions

**DOI:** 10.1038/s41597-022-01529-6

**Published:** 2022-07-18

**Authors:** Kevin Spiekermann, Lagnajit Pattanaik, William H. Green

**Affiliations:** Department of Chemical Engineering, Massachusetts Institute of Technology, 77 Massachusetts Ave, Cambridge, MA 02139 USA

**Keywords:** Chemical engineering, Cheminformatics, Quantum chemistry

## Abstract

Quantitative chemical reaction data, including activation energies and reaction rates, are crucial for developing detailed kinetic mechanisms and accurately predicting reaction outcomes. However, such data are often difficult to find, and high-quality datasets are especially rare. Here, we use CCSD(T)-F12a/cc-pVDZ-F12//*ω*B97X-D3/def2-TZVP to obtain high-quality single point calculations for nearly 22,000 unique stable species and transition states. We report the results from these quantum chemistry calculations and extract the barrier heights and reaction enthalpies to create a kinetics dataset of nearly 12,000 gas-phase reactions. These reactions involve H, C, N, and O, contain up to seven heavy atoms, and have cleaned atom-mapped SMILES. Our higher-accuracy coupled-cluster barrier heights differ significantly (RMSE of ∼5 kcal mol^−1^) relative to those calculated at *ω*B97X-D3/def2-TZVP. We also report accurate transition state theory rate coefficients ${k}_{\infty }(T)$ between 300 K and 2000 K and the corresponding Arrhenius parameters for a subset of rigid reactions. We believe this data will accelerate development of automated and reliable methods for quantitative reaction prediction.

## Abstract (summary)

**Table:** 

Measurement(s)	Barrier Heights • Enthalpies • Rate Coefficients
Technology Type(s)	ab initio quantum chemistry computational method

## Background & Summary

Detailed reaction mechanisms are valuable tools for analyzing and predicting physical phenomena driven by chemical kinetics. Historically, kinetic model parameters were fit to a specific set of experimental results, which limited their generalizability to systems with different temperatures, pressures, or initial compositions. In recent decades, the field of chemical kinetics has transitioned from postdictive to predictive modeling approaches^1^. This shift has been motivated by advances in compute power, which make it possible to predict many kinetic parameters using ab initio calculations rather than relying on scarce experimental data^2–13^. Our research group has long been interested in the automated generation of kinetic models, which can simulate and predict the concentrations of all relevant species^14,15^. Reliable datasets are essential for constructing such models with predictive power. A small error of a few kcal mol^−1^ in the activation energy will lead to significant errors in the final rate estimate, particularly at lower temperatures. Unfortunately, accurate barriers are often known for fewer than 10% of the reactions in kinetic models^8–13,16,17^. To help address this paucity of data, here we present relatively accurate barriers for nearly 12,000 reactions.

Kinetic parameters are currently estimated using functional group and linear-free-energy (LFER) methods,^18,19^, but machine learning models are much more flexible and have broader scope. Indeed, machine learning has sparked an explosion of progress in physical and organic chemistry, especially in the areas of automated synthesis planning^20,21^, targeted molecular optimization^22,23^, and general property prediction^24,25^ from thermodynamic^26,27^ and solvation parameters^28,29^ to full infrared spectra^30^. In situations where data are plentiful, machine learning-based algorithms often provide excellent predictions and some have successfully been applied to experiments^31^. When such data is lacking, researchers generate their own datasets–both experimentally and computationally–to regress desired properties from them^32–34^. In machine learning applied to chemistry, the community has largely taken a model-driven approach, where significant effort has been devoted to refining models on a few benchmark datasets^35,36^. As a result, the community has delivered strong architectures from advanced graph convolutional networks^37–39^ to atomistic networks^40,41^. Today, progress is limited primarily by the scarcity of large, diverse, and high-quality datasets.

Here, we report a cleaned, high-quality dataset of reaction barriers, enthalpies, and transition state theory (TST) rate coefficients. We build upon the prior work from Grambow *et al*^42,43^. Briefly, their work used the single-ended growing string method^44^ to automatically identify thousands of transition states (TSs) and products from a given set of reactants. Reactants were chosen by using all molecules with six or fewer heavy atoms from GDB-7^45^ as well as randomly selecting some (∼430) molecules with seven heavy atoms; the molecules contain H, C, N, and O atoms. Conformer searches were performed for the reactants by embedding several hundred conformers for each molecule using RDKit^46^ with the ETKDG distance geometry method^47^ and relaxing their geometries using the MMFF94 force field implemented in RDKit. The lowest energy conformer was then optimized using Q-Chem^48^ at both the B97-D3/def2-mSVP level of theory with Becke-Johnson damping^49^ and the *ω*B97X-D3/def2-TZVP^50^ level of theory. The reactant conformer was the starting point for the growing string search. The highest energy point in the string was used as the initial guess for a conventional saddle point search. Additional details can be found in the original publication.

Our work brings the following advances. First, we clean the SMILES^51^ reported in Grambow *et al*^42^. The original publication treated all reactions with multiple products as containing one product complex, which does not conform well with traditional TST calculations that expect partition functions for each species. Thus, we separate the products from any product complex, recalculate the geometry optimization and frequency at either B97-D3/def2-mSVP or *ω*B97X-D3/def2-TZVP. Finally, we refine the single point energies for each species using explicitly correlated coupled-cluster calculations, which are expected to be much more accurate than the density functional theory methods^52–55^. We provide the updated barrier heights and reaction enthalpies from our CCSD(T)-F12a/cc-pVDZ-F12//*ω*B97X-D3/def2-TZVP calculations. Our higher-accuracy calculations improve the RMSE of the barrier heights by approximately 5 kcal mol^−1^ relative to those calculated at *ω*B97X-D3/def2-TZVP. We believe that the high-quality values in this dataset will accelerate development of automated and reliable methods for quantitative reaction prediction. Finally, we also identify a subset of reactions with rigid species that do not require a conformer search nor hindered-rotor treatment. The rigid-rotor harmonic oscillator (RRHO) TST rate coefficients ${k}_{\infty }(T)$ and fitted Arrhenius parameters for this subset are reported since these values should be accurate. We do not report ${k}_{\infty }(T)$ or Arrhenius parameters for reactions involving flexible reactants or transition states since RRHO TST is not accurate for these reactions.

## Methods

### Overview

Dataset refinement started by cleaning the SMILES from the original dataset^43^ and filtering reactions to those containing one reactant and at most three products. Next, product complexes are separated into individual species, each of which is reoptimized at the respective level of theory i.e. either B97-D3/def2-mSVP or *ω*B97X-D3/def2-TZVP. The single point energy of all species optimized at *ω*B97X-D3/def2-TZVP is computed at CCSD(T)-F12a/cc-pVDZ-F12. These energies are used to calculate updated barrier heights by adding the zero-point energies (ZPEs) from the harmonic vibrational analysis to the reactant, product, and TS energies and then computing the difference between the resulting TS and reactant energies. Similarly, enthalpies of reaction are calculated based on the difference of the ZPE-corrected product and reactant energies; bond additivity corrections (BACs) are added to each species. Finally, we identify a subset of reactions that contain rigid species, calculate high-pressure limit TST rate coefficients, and report the fitted Arrhenius parameters.

### Cleaning SMILES

**Fig. 1:**
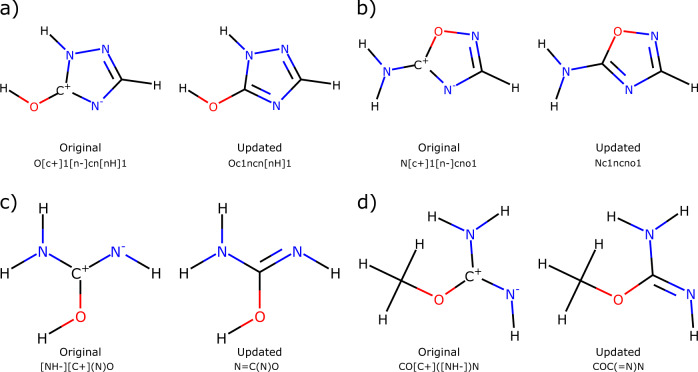
Representative examples of updating SMILES to correspond to the most representative resonance structure. For clarity, the SMILES shown here omit atom-map numbers.

**Fig. 2:**
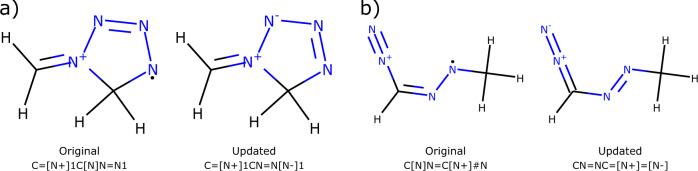
Representative examples of updating SMILES to fix incorrect charge imbalances. For clarity, the SMILES shown here omit atom-map numbers.

The work from Grambow *et al*.^42^ used the single-ended growing string method^44^ to generate a list of possible products from a given reactant. The input and output for the growing string method are a set of three-dimensional coordinates to describe the molecule or multi-molecule complex. Grambow *et al*. used Open Babel^56^ to perceive connectivity and generate a SMILES for the reactant and product from each set of three-dimensional coordinates. However, in some cases, the bond-order and formal charges did not correspond to the most representative resonance structure. Here, we update the SMILES by using RDKit^46^ to look for neighboring atoms with opposite formal charges, which often occurred between nitrogen and carbon atoms. Some representative examples of the updated SMILES and their impact on molecular structure are shown in Fig. 1. Additionally, there were a handful of reactions whose reactant was neutral, but whose product was positively charged. This charge imbalance was likely due to Open Babel occasionally generating an incorrect SMILES from the molecular coordinates. Here, we update the corresponding product SMILES to conserve charge for the reaction i.e. added an electron to create a correct Lewis structure. Representative examples are shown in Fig. 2. Atom-mapping is preserved when updating the SMILES.

### Reoptimizing products

Of the reactions in the previously published dataset from Grambow *et al*.^42^, approximately 30% contain two products and 2% contain three products. These were previously treated as one product complex when using the growing string method as well as during subsequent geometry optimization and frequency calculation. However, to obtain rate coefficients, conventional canonical TST calculations expect partition functions for each individual species. Here, we separate the complexes into individual products, reoptimize the geometries, and recalculate the frequencies using Q-Chem 5.3.0^48^ for both the B97-D3/def2-mSVP and *ω*B97X-D3/def2-TZVP datasets. The previously optimized geometries from the complex are used as the initial guess for the new optimization. The exact same settings are used in the input files as were used by Grambow *et al*.^42^ during the original Q-Chem calculations. This ensures that the separated products are run with the same method and basis set as well as with identical convergence criteria as those used by their corresponding reactant and TS.

Consistent with the previous work, nearly all molecules are run in the singlet state and use a spin-unrestricted ansatz. For example, the ground electronic state for methylene (CH_2_) is a triplet, but because the TS for all reactions was computed at the singlet state, any CH_2_ products were also recalculated in the singlet state. However, upon splitting some products, there are 49 reactions unique to the larger B97-D3 dataset whose product pairs were radicals; these individual species were calculated in the doublet state since it was assumed that the lone electron on each product had opposite spins to conserve the overall multiplicity for the reaction. We verified that spin contamination is not a problem by confirming that the average value of the total spin operator is between 0.75 and 0.77 for these species. The value of <S^2^> is 0 for all other species.

Note that reoptimizing the separated products and then summing their energy resulted in a different product energy than that from the original product complex. In a few cases, this changed the reaction enthalpy enough such that ΔH > ΔE_0_, which would cause the reverse reaction to have a negative barrier height. Although submerged barriers are possible, often a large negative barrier height is reason to be suspicious. Thus, we remove any reaction in which the explicitly correlated coupled-cluster reaction enthalpy was more than 10 kcal mol^−1^ larger than the barrier height.

### Refining single point energies

A major accomplishment of this work is providing highly accurate kinetic parameters computed at RCCSD(T)-F12a/cc-pVDZ-F12//*ω*B97X-D3/def2-TZVP for a large and diverse set of atom-mapped gas phase reactions. All species were calculated in the singlet state. Although the *ω*B97X-D3 method is more accurate for predicting barrier heights than many other functionals^57^, coupled-cluster CCSD(T) calculations are commonly considered the gold-standard in quantum chemistry^58,59^. Here, we refine the single point energies of each species from the *ω*B97X-D3/def2-TZVP dataset using the explicitly correlated CCSD(T)-F12 method since previous literature has shown that CCSD(T)-F12 can achieve similar accuracy to the standard CCSD(T) calculation while using a much smaller basis set^3,52–55,60^. This is notable because both coupled-cluster methods scale as $O({N}^{7})$, such that *N* is the number of orbitals^61^, so using a smaller basis set offers substantial computational savings.

We next consider which basis set to use. Although triple-$\zeta$ and quadruple-$\zeta$ basis sets have shown reaction energies within 1 kcal mol^−1^, many studies comparing basis sets use about 100 molecules or fewer. Further, such studies often focus on small molecules containing primarily three or four heavy atoms due to the steep scaling of coupled-cluster calculations. The main exception to this generalization is the recent calculation of the 133,000 molecules from QM9^35^ with the G4MP2 level of theory^62,63^; however, that dataset only contains stable species, while our dataset has approximately 12,000 TSs. Considering that our dataset contains nearly 22,000 unique stable species and transition states, each containing up to seven heavy atoms, we chose the cc-pVDZ-F12 basis set to accommodate the large number of calculations while maintaining high accuracy. 15 reactions were also run with cc-pVTZ-F12 to validate the double-$\zeta$ accuracy. For all coupled-cluster single point calculations, we use the energy from CCSD(T)-F12a since both published literature^64^ and the MOLPRO documentation^65^ conclude this method offers a better approximation to the complete basis set limit for the double-$\zeta$ and triple-$\zeta$ basis sets. All calculations were run in parallel using MOLPRO 2015.1^65^ on the National Energy Research Scientific Computing Center (NERSC).

### Calculating reaction barrier heights and enthalpies

ZPEs from the harmonic vibrational analysis are added to the electronic energy for the reactant, product, and TS. For all species, a scaling factor is applied to the computed harmonic frequencies used to compute the ZPE; the scaling factor for B97-D3/def2-mSVP and for *ω*B97X-D3/def2-TZVP are calculated as described by Alecu *et al*.^66^ and found to be 1.014 and 0.984 respectively. Reaction barrier heights are computed by taking the difference of the resulting TS and reactant energies. Similarly, enthalpies of reaction at 298 K are computed by taking the difference of the resulting product and reactant energies. When calculating the values for the coupled-cluster dataset, the CCSD(T)-F12a energies are used while the ZPEs are taken from the *ω*B97X-D3 calculation since this was the level of theory used for the geometry optimization and vibrational analysis. Note that atom energy corrections (AECs) and bond additivity corrections (BACs) are added to the enthalpy values for each species. Although the AECs cancel out during the subtraction to obtain the reaction enthalpy since all reactions are balanced, these corrections are important when comparing the ${\Delta }_{{\rm{f}}}$H(298) to experimental values as described in the technical validation. Corrections are not used when computing reaction barriers. AECs are calculated by fitting the atomization energies of 14 small molecules. The atomization energies come from CCCBDB^67^ and all have uncertainty values less than 0.2 kcal mol^−1^. Petersson type BACs^68^ were fit using a set of about 400 reference species with well-known heats of formation, primarily drawn from ATcT^69^ and CCCBDB^67^. The experimental uncertainty is at most 0.55 kcal mol^−1^, though most values are much lower with the median being just 0.14 kcal mol^−1^. For more details on the fitting procedure, see the Reaction Mechanism Generator (RMG) documentation at https://reactionmechanismgenerator.github.io/RMG-Py/users/arkane/input.html#atom-energy-fitting.

### Calculating rates

Automated Reaction Kinetics and Network Exploration (Arkane) is a software package for computing thermodynamic properties and high-pressure limit rate coefficients using the results from quantum chemistry calculations. Thermodynamic properties are computed using the RRHO approximation, while kinetic parameters are computed using conventional canonical TST, also with RRHO. Arkane is developed and distributed as part of RMG-Py^14,15^. All software is written in Python and provided as free, open source code under the terms of the MIT License.

We use Arkane to convert the single point energy from the quantum chemistry calculation to the gas-phase reference state; by default atom and spin-orbit coupling energy corrections are applied, but will cancel during the TST calculation. As before, the corresponding scaling factor is applied to the ZPE for each species. Arkane uses RRHO TST with Eckart tunneling correction to calculate the forward rate coefficient for a set of user-defined temperatures. BACs are omitted when calculating the forward rate coefficient since BACs are not present for the partial bonds in the TS. Arkane then uses a linear least-squares fitting to fit the list of reciprocal temperatures and logarithm of the rate coefficients to an Arrhenius expression, yielding the best approximation for the pre-exponential A-factor and the activation energy. We use 50 linearly spaced points in the reciprocal temperature space between 300 K and 2000 K when obtaining the Arrhenius parameters.

## Data Records

All data is free and publicly accessible on Zenodo^70^. Q-Chem output files are provided for the 16,302 reactions at B97-D3/def2-mSVP and for the 11,926 reactions at *ω*B97X-D3/def2-TZVP level of theory. For convenience, these also include the original log files for the reactant, TS, and non-reoptimized products from Grambow *et al*.^42^ since they are used to calculate barrier heights, enthalpies, and rate coefficients in this work. MOLPRO output files from the single point calculations are provided for the 11,926 reactions at the CCSD(T)-F12a/cc-pVDZ-F12 level of theory as well as for the 15 reactions calculated with the triple-$\zeta$ basis. Information for each reaction is organized by the level of theory and stored in a separate folder labeled as rxn######, such that ###### denotes the reaction number padded with zeros. The numbering matches that from the originally published dataset^43^ to facilitate easy comparison. For the quantum chemistry calculations, each folder contains the log files for the reactant, TS, and product as r######.log, ts######.log, and p######.log respectively. An additional number is appended to the file names from the separated products. For example, the log files for any reaction containing two products are labeled as p######_0.log and p######_1.log.

The cleaned atom-mapped SMILES, as well as all values calculated in this work, are provided in the comma-separated values (csv) files b97d3.csv, wb97xd3.csv, ccsdtf12_dz.csv, and ccsdtf12_tz.csv. The columns for the csv files are described in Table 1. The calculated TST rate coefficients and fitted Arrhenius parameters for the rigid species are provided in ccsdtf12_dz_rigid.csv, whose columns are described in Table 2. The Arkane output files from TST calculations and Arrhenius parameter fitting are also provided. Each reaction is again stored in a separate folder labeled as rxn######, which contains a rxn folder with all information from the Arrhenius fitting. The kinetic information is stored in Chemkin^71^ file format. The list of 50 temperatures (K) used during Arrhenius fitting is provided in arkane_temperatures.csv.

**Table 1:** Description of the columns in the main comma-separated value files for each level of theory.

Column label	Description
idx	Reaction index
rsmi	Reactant SMILES
psmi	Product SMILES
rinchi	Reactant InChI
pinchi	Product InChI
dE0	Barrier height $({\text{kcal mol}}^{-1})$
dHrxn298	Enthalpy of reaction $({\text{kcal mol}}^{-1})$
rmg_family	RMG reaction family

**Table 2:** Description of the columns in the comma-separated value file for rigid reactions.

Column label	Description
idx	Reaction index
rsmi	Reactant SMILES
psmi	Product SMILES
k(T0) to k(T49)	50 columns with the calculated rate coefficient $\left({{\rm{s}}}^{-1}\right)$
lnA	Natural log of the fitted pre-exponential factor $\left({{\rm{s}}}^{-1}\right)$
Ea	Fitted activation energy $({\text{kcal mol}}^{-1})$
percent_error	Average absolute percent error between the calculated and fitted rate coefficients

The improvement from fitting BACs at B97-D3/def2-mSVP, *ω*B97X-D3/def2-TZVP, CCSD(T)-F12a/cc-pVDZ-F12//*ω*B97X-D3/def2-TZVP, and CCSD(T)-F12a/cc-pVTZ-F12//*ω*B97X-D3/def2-TZVP is contained in b97d3_def2msvp_BAC.csv, wb97xd3_def2tzvp_BAC.csv, ccsdtf12_ccpvdzf12__wb97xd3_def2tzvp_BAC.csv, and ccsdtf12_ccpvtzf12__wb97xd3_def2tzvp_BAC.csv respectively. The files contain the experimental and calculated enthalpies for the reference species from RMG-database used for fitting. The atom and bond correction values are publicly stored on the RMG-database GitHub, though they are also provided in fitted_corrections.pkl for convenience. Further validation of the BACs at the double-$\zeta$ basis set is done by comparing to experimental values from the Pedley^72^ set since over half of these molecules are not in the RMG-database training set used for fitting. The comparison is shown in ccsdtf12_dz_vs_Pedley_experimental.csv.

## Technical Validation

**Fig. 3:**
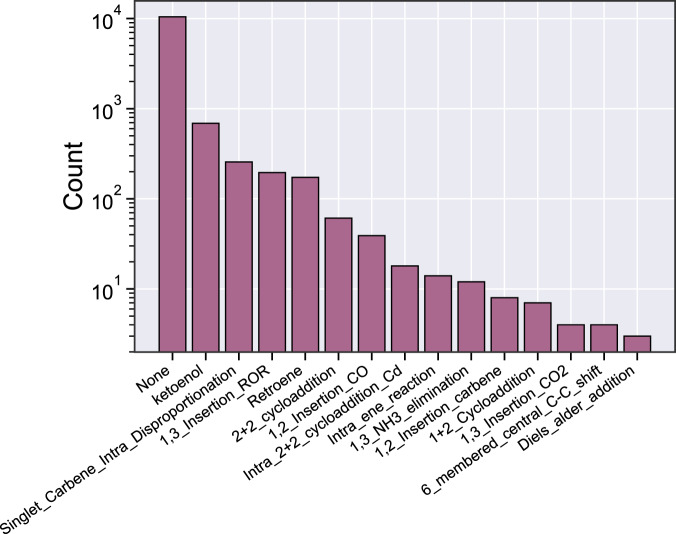
Distribution of RMG reaction families present in the CCSD(T)-F12a/cc-pVDZ-F12 dataset.

The published work from Grambow *et al*.^42^ already performed several integrity checks, such as ensuring that all TSs have exactly one imaginary frequency, whose atomic displacements matched the bond changes occurring between the reactant and product. The authors also removed any TS with an imaginary frequency less than 100 cm^−1^ in magnitude as that typically corresponds to conformational changes. In this work, we ensure that multiplicity and charge are conserved for all reactions. As described in the methods section, this is important when separating product complexes into individual product geometries for reoptimization. We next identify whether each reaction matches a reaction template from the RMG-database. As shown in Fig. 3, keto-enol is the most represented RMG template. However, due to the diversity of these reactions, the majority do not match any RMG template. This is consistent with the previously published work^42^, which chose to characterize the reaction diversity by extracting general templates that do not necessarily match a template from RMG-database. RMG-database is frequently updated, which includes occasionally updating the reaction templates to be more broad or more specific. Thus, using reaction templates from a different version of RMG-database may capture more or less reactions for a given family (or even include different reaction families). To generate Fig. 3 and Table 3, we used the AEC_BAC branch of RMG-database.

**Table 3:** RMG reaction templates present in the CCSD(T)-F12a/cc-pVDZ-F12 dataset.

RMG Reaction Family	Template
ketoenol	
Singlet_Carbene_Intra_Disproportionation	
1,3_Insertion_ROR	
Retroene	
2+2_cycloaddition	
1,2_Insertion_CO	
Intra_2+2_cycloaddition_Cd	
Intra_ene_reaction	
1,3_NH3_elimination	
1,2_Insertion_Carbene	
1+2_Cycloaddition	
1,3_Insertion_CO2	
6_membered_central_C-C_shift	
Diels_alder_addition	

To evaluate the improvement from applying the fitted atom and bond corrections to the enthalpy values, we calculate the error relative to the high-quality reference set of about 400 molecules used for fitting. For the coupled-cluster data, the training mean absolute error (MAE) and root mean squared error (RMSE) are 0.5 and 0.8 kcal mol^−1^ respectively. We also compare the corrected enthalpy values to an external test set originally published by Pedley^72^ and compiled and verified by Narayanan *et al*.^62^ to validate our enthalpy calculation approach (CCSD(T)-F12a + AEC + BAC). This set, named the Pedley test set, contains 459 species that have experimental uncertainty, defined as 95% confidence intervals^73,74^, of less than 1 kcal mol^−1^. After removing 76 species common to both our reference set and our coupled-cluster dataset (so as to exclude training species from our test set), we measure the error of our corrected enthalpies against the Pedley test set. This evaluation returns an MAE of 0.8 kcal mol^−1^ and RMSE of 1.2 kcal mol^−1^, indicating strong agreement of our approach with high-quality experimental data.

**Fig. 4:**
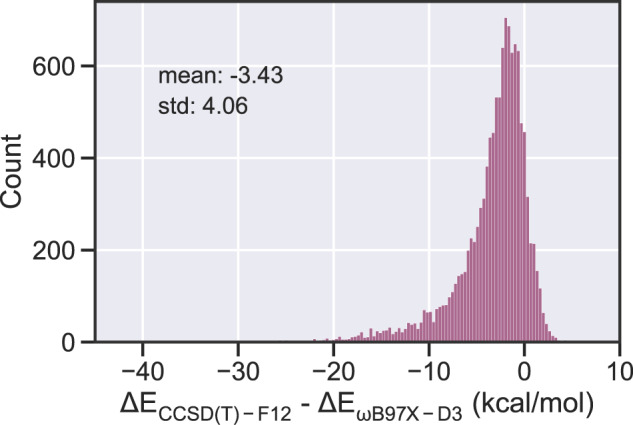
Difference in barrier height calculated at CCSD(T)-F12a/cc-pVDZ-F12//*ω*B97X-D3/def2-TZVP and *ω*B97X-D3/def2-TZVP.

To compare accuracy improvements from the coupled-cluster calculations in this work, Table 4 shows the MAE and RMSE of the barrier height and reaction enthalpy relative to the lower levels of theory. The summary statistics are calculated using the list of about 10,400 reactions that are common to all three level of theory datasets. As expected, values from the *ω*B97X-D3 dataset show less deviation than those from the B97-D3 dataset, yet they are still several kcals away from the explicitly correlated coupled-cluster values. The RMSE of 5 kcal mol^−1^ is significant since it implies that rate coefficients calculated at *ω*B97X-D3 differ on average by a factor of 12 at 1,000 K relative to those calculated at CCSD(T)-F12a; the difference increases substantially to a factor of 4,000 at 300 K. It is interesting to note that the RMSE for barrier heights reported here is more than twice as large as the RMSE reported in Ref. ^57^. However, this previous analysis was done with 206 reactions from just a few reaction families, whereas the data presented in Table 4 represent more than 10,000 reactions and a much more diverse array of chemistry. Taking the barrier height RMSE of only RMG reaction families gives 8.0 and 3.8 kcal mol^−1^ for the B97-D3 and *ω*B97X-D3 dataset respectively, both of which are smaller deviations than that for the entire dataset. As seen in Fig. 4, the barrier heights calculated at DFT tended to be an overestimate relative to those calculated at CCSD(T)-F12a/cc-pVTZ-F12//*ω*B97X-D3/def2-TZVP. On average, the difference is a few kcal mol^−1^, though there are a minority of reactions in which the errors are larger. Further exploration as to why this DFT functional gives such different values could be an area of future research.

**Table 4:** Errors in ${\text{kcal mol}}^{-1}$ for each level of theory relative to CCSD(T)-F12a/cc-pVDZ-F12.

Level of Theory	Barrier Height		Reaction Enthalpy	
	MAE	RMSE	MAE	RMSE
B97-D3/def2-mSVP	7.0	8.5	3.5	4.8
*ω*B97X-D3/def2-TZVP	3.5	5.0	1.8	2.5

We next compare some of the coupled cluster values in our dataset to those from other published works. For example, Dontgen *et al*.^75^ reported ten keto-enol reactions calculated at DLPNO-CCSD(T)/CBS//B3LYP-D3BJ/def2-TZVP. Two of their reactions (reactions 1 and 7) are also present in our dataset. For both reactions, the barrier heights agree within 0.3 kcal mol^−1^. Balabin^76^ calculated tautomerization reactions of triazoles at CCSD(T)/CBS//MP2/aug-cc-pVT. For the reaction of 1H-1,2,3-triazole producing 2H-1,2,3-triazole, they report a reaction enthalpy of −3.98 kcal mol^−1^ compared to our calculated value of −3.96 kcal mol^−1^. Their reported barrier height for the reverse direction is 53.6 kcal mol^−1^ compared to our value of 49.8 kcal mol^−1^, calculated by subtracting our reaction enthalpy from our barrier height for the forward direction.

To further evaluate the improvement from the double-$\zeta$ single point calculations, we also calculate some reactions at CCSD(T)-F12a/cc-pVTZ-F12//*ω*B97X-D3/def2-TZVP. The triple-$\zeta$ calculations required substantially more computational time and scratch space when compared to the double-$\zeta$ calculations. Three reactions are sampled from each of the top five most common RMG reaction families, shown in Fig. 3. When looking at the distribution of barrier heights within each family for the double-$\zeta$ basis set, the three reactions are chosen to represent approximately the 25^th^, 50^th^, and 75^th^ percentile. Table 5 and Table 6 show the MAE and RMSE of the barrier height and reaction enthalpy respectively from the different levels of theory with respect to the triple-$\zeta$ basis set. These trends are consistent with other previous literature that emphasizes the high fidelity of explicitly correlated coupled-cluster calculations, even with a double-$\zeta$ basis set^55,60^.

**Table 5:** Barrier height errors $({\text{kcal mol}}^{-1})$ for each level of theory relative to CCSD(T)-F12a/cc-pVTZ-F12 for sample reactions.

Reaction Family	B97-D3/def2-mSVP		*ω*B97X-D3/def2-TZVP		CCSD(T)-F12a/cc-pVDZ-F12	
	MAE	RMSE	MAE	RMSE	MAE	RMSE
1,3 Insertion ROR	7.6	7.6	0.7	0.8	0.06	0.08
2+2 Cycloaddition	9.0	9.0	2.5	2.5	0.06	0.09
Keto-Enol	4.8	5.7	0.9	1.4	0.05	0.05
Retroene	14.7	15.1	1.4	1.5	0.24	0.24
Singlet Carbene Intra Disproportionation	0.5	0.5	0.3	0.4	0.04	0.05
**Overall**	7.3	9.0	1.2	1.5	0.09	0.12

**Table 6:** Reaction enthalpy errors $({\text{kcal mol}}^{-1})$ for each level of theory relative to CCSD(T)-F12a/cc-pVTZ-F12 for sample reactions.

Reaction Family	B97-D3/def2-mSVP		*ω*B97X-D3/def2-TZVP		CCSD(T)-F12a/cc-pVDZ-F12	
	MAE	RMSE	MAE	RMSE	MAE	RMSE
1,3 Insertion ROR	1.2	1.4	0.7	0.8	0.04	0.04
2+2 Cycloaddition	1.6	1.8	1.5	1.6	0.13	0.14
Keto-Enol	2.7	2.9	0.6	0.7	0.12	0.12
Retroene	6.4	6.6	1.5	1.6	0.16	0.18
Singlet Carbene Intra Disproportionation	5.1	5.3	2.9	3.0	0.11	0.12
**Overall**	3.4	4.1	1.4	1.8	0.11	0.13

**Fig. 5:**
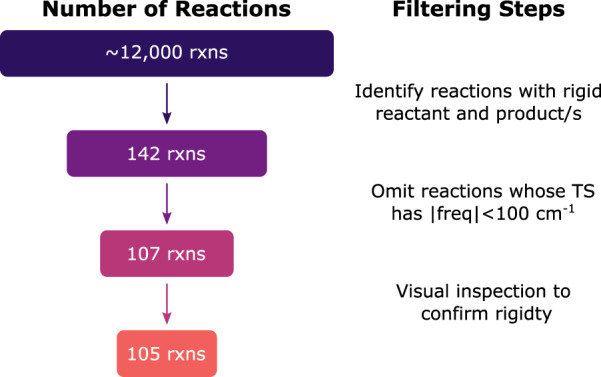
Schematic workflow for identifying rigid reactions.

**Fig. 6:**
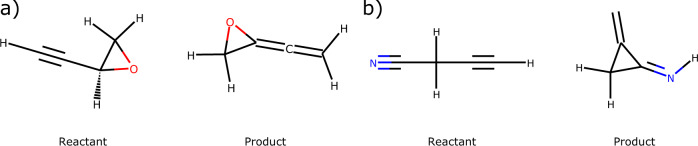
Examples of rigid reactions (**a**) rxn001645 (**b**) rxn002603.

Finally, Grambow *et al*.^42^ already performed a conformer search for the reactants. No additional conformer searching is done in this work. Instead, we identify rigid reactions that do not require a conformer search and report the RRHO TST rate coefficients and fitted Arrhenius parameters for this subset since the explicitly correlated coupled-cluster values should be quite reliable. Fig. 5 summarizes the filtering workflow to identify rigid reactions. We start by using RDKit’s Lipinski rotatable bond SMARTS to find reactions whose reactant and product(s) do not have any rotatable bonds. Further, if any rings are present, we filter molecules with only planar rings (either aromatic or 3-membered). With these criteria, we identify a subset of reactions from the CCSD(T)-F12a/cc-pVDZ-F12 dataset that contain rigid stable species. We next omit any reaction whose TS has a positive frequency smaller than 100 cm^−1^ since this is a common threshold for distinguishing conformational motions from vibrational modes that will be used in the rigid rotor harmonic oscillator approximation^77–80^. As the last filtering step, we visually inspect the remaining reactions to verify that RDKit correctly identified rigid species and also confirm that the TS would not require a conformer search either; this left 105 rigid reactions. Two examples are shown in Fig. 6.

**Fig. 7:**
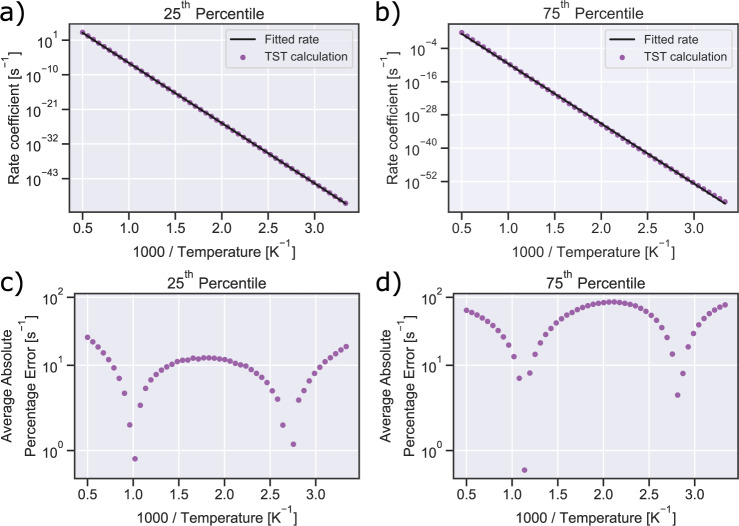
Arkane fitting for rigid reactions corresponding to (**a**) the 25^th^ percentile (rxn001645) and (**b**) the 75^th^ percentile (rxn002603) of average absolute percentage error from the CCSD(T)-F12a/cc-pVDZ-F12 dataset. Residuals between the TST rate coefficients and those calculated from the Arrhenius fit are shown in (**c**) and (**d**).

To determine whether the fitted parameters give a good estimate of the rate coefficient for these rigid reactions, we examine the average absolute percentage error between the rate coefficient calculated from TST and the predicted rate coefficients using the fitted parameters. The largest value is 233% i.e. about a factor of 3 error in the rate coefficient which is often quite acceptable, though most reactions have a much lower error. For instance, 62 of these rigid reactions have average fitting errors below 20%; thus, the fitted A-factor and activation energy should be very reliable for these reactions. Residuals from the least-squares fit for reactions with the 25^th^ and 75^th^ percentile for average percentage error are shown in Fig. 7. For nearly all data points, the residuals are very close to zero. Lastly, we searched for published experimental data to compare with our calculated values. Saito *et al*.^81^ studied isomerization of acetonitrile to methyl isocyanide at 1600–2100 K behind reflected shock waves. They report ${k}_{\infty }(T)=1{0}^{13.5}$ exp(−260 kJ mol^−1^/RT) s^−1^, which agrees quite well with the A-factor of 1.14 $\times 1{0}^{14}$ s^−1^ and Ea of 262 kJ mol^−1^ from our fitted Arrhenius expression.

## Usage Notes

Except for the commercial Q-Chem and MOLPRO quantum chemistry softwares, all code necessary to reproduce the generated data is available on GitHub^82^. The repository contains scripts, which should be run in the following order:

- Jupyter notebooks were used to identify potentially erroneous SMILES for the reactants and products of both the B97-D3/def2-mSVP and *ω*B97X-D3/def2-TZVP level of theory. The suggested SMILES were manually inspected, utilizing the interactive nature of Jupyter notebooks, to confirm that the change was chemically reasonable and preserved the atom-mapping.
- create_qchem_input_files.py: Parses the original Q-Chem log files from Grambow *et al*.^42^ to separate product complexes into the individual Q-Chem input files for both the B97-D3/def2-mSVP and *ω*B97X-D3/def2-TZVP levels of theory.
- create_molpro_input_files.py: Creates MOLPRO input files for the single point calculations at CCSD(T)-F12a/cc-pVDZ-F12 using the reoptimized *ω*B97X-D3/def2-TZVP geometries.
- parse_barriers_enthalpies.py: Compiles the reactant and product SMILES into comma-separated values files and parses the reaction barrier heights and enthalpies.
- get_enthalpies_corrected.py: Uses the atom and bond corrections from RMG-database to obtain more accurate reaction enthalpies.
- identify_rmg_reactions.py: Identifies which reactions correspond to RMG reaction templates.
- identify_rigid_species.py: Uses RDKit to identify reactions with rigid reactant and product.
- run_arkane.py: Runs Arkane to obtain Arrhenius rate parameters for the rigid reactions.
- parse_tst_rates.py: Parses the calculated rate coefficients from the Arkane output files.
- parse_arrhenius_parameters.py: Parses the fitted A-factors and activation energies from Arkane output files.
- calculate_percent_error.py: Calculates the average percent error between the rate coefficients calculated from TST and those predicted using the fitted Arrhenius parameters.

## Data Availability

The code used to generate this data is freely available on GitHub under the MIT license^82^. Details on how to use the scripts to generate the data are provided in the Usage Notes. Some of the scripts utilize helpful components of the Reaction Mechanism Generator, such as RMG-Py, RMG-database, and the Automatic Rate Calculator (ARC)^83^. All related software is open-source under the MIT license and freely accessible on GitHub. For RMG-Py, checkout the qchem_parser branch, and for RMG-database, checkout AEC_BAC. The GitHub version commit string was ea2eb625fb1dcc6892ef6ddd5d7fdc96abf477e1 for ARC on the main branch.
